# CD163-positive macrophage enrichment is associated with mismatch repair deficiency and adverse pathologic features in early-stage endometrioid endometrial carcinoma: a retrospective cohort study

**DOI:** 10.3389/fonc.2026.1954599

**Published:** 2026-10-02

**Authors:** Donglian Chen, Xiaojuan Huang, Yu Zhang, Linhong Liao, Qianqian Ye, Yu Guo

**Affiliations:** 1 Department of Pathology, Ganzhou Maternal and Child Health Hospital, Ganzhou, China; 2 Department of Obstetrics and Gynecology, Ganzhou Maternal and Child Health Hospital, Ganzhou, China

**Keywords:** CD163, disease-free survival, endometrioid endometrial carcinoma, mismatch repair deficiency, tumor-associated macrophage

## Abstract

**Background:**

Mismatch repair deficiency defines an immunologically distinct subgroup of endometrioid endometrial carcinoma, but its relationship with CD163-positive macrophage infiltration in early-stage disease remains incompletely characterized. We evaluated the association between MMR status and quantitative CD163-positive TAM infiltration, together with secondary clinicopathologic and exploratory DFS analyses.

**Methods:**

This single-center retrospective cohort included patients with surgically treated FIGO 2009 stage I–II endometrioid endometrial carcinoma and available formalin-fixed paraffin-embedded tumor tissue. Immunohistochemistry for MLH1, PMS2, MSH2, MSH6, and CD163 defined MMR status and quantified macrophage density. CD163-positive TAM density was analyzed quantitatively as the principal measure, with a cohort-median low/high categorization retained for secondary phenotypic analyses. Median regression evaluated the association between MMR status and continuous CD163-positive TAM density, whereas logistic regression was used for the secondary binary TAM analysis and the composite adverse pathologic endpoint. Exploratory DFS analyses included single-marker and combined Cox models, formal MMR-by-TAM interaction testing, and comparison of models with and without continuous CD163-positive TAM density.

**Results:**

Among 254 patients assessed, 236 were included. MMR protein expression was retained in 172 tumors (72.9%), whereas 64 (27.1%) were classified as dMMR. CD163-positive TAM density was higher in dMMR than pMMR tumors (median, 76 vs 47 cells/HPF; P<0.001), and high TAM infiltration was more frequent in dMMR tumors (44/64, 68.8% vs 71/172, 41.3%; P<0.001). After adjustment for age, FIGO stage, tumor grade, tumor size, and deep myometrial invasion, dMMR remained associated with high macrophage infiltration (adjusted OR, 2.27; 95% CI, 1.19–4.18; P = 0.008). High macrophage infiltration was associated with grade 3 histology, deep myometrial invasion, LVSI, and the composite adverse pathologic feature; the association with the composite endpoint persisted after adjustment (adjusted OR, 1.78; 95% CI, 1.01–3.14; P = 0.047). During follow-up, 26 DFS events occurred. In exploratory Cox analyses, dMMR was associated with DFS (adjusted HR, 2.76; 95% CI, 1.25–6.10; P = 0.012), whereas continuous CD163-positive TAM density was not (adjusted HR per 10 cells/HPF, 1.04; 95% CI, 0.97–1.12; P = 0.281), and addition of CD163-positive TAM density did not significantly improve model fit beyond MMR status (likelihood-ratio P = 0.279). The dMMR/high-TAM phenotype retained an exploratory association with DFS in the conventional model (adjusted HR, 2.21; 95% CI, 1.02–4.81; P = 0.044), but the association was attenuated after additional adjustment for adjuvant therapy (HR, 2.04; 95% CI, 0.93–4.52; P = 0.075).

**Conclusions:**

IHC-defined dMMR was associated with increased CD163-positive macrophage infiltration, and higher TAM infiltration was associated with adverse pathologic features. DFS findings were exploratory and did not establish incremental prognostic value of CD163-positive TAM infiltration beyond MMR status; larger externally validated cohorts are required to assess prognostic relevance.

## 1 Introduction

Endometrioid endometrial carcinoma is commonly diagnosed at an early stage and is generally associated with favorable survival after surgery (1–4). In routine clinical practice, postoperative risk assessment for early-stage disease is still largely based on conventional pathological variables, including FIGO stage, tumor grade, depth of myometrial invasion, and lymphovascular space invasion (5, 6). These variables remain indispensable for treatment planning and follow-up intensity, but they do not fully explain why some patients with apparently localized disease develop adverse pathological profiles or subsequent recurrence (1, 3, 7). This limitation is particularly relevant in real-world surgical cohorts, where most patients are treated with primary hysterectomy and risk stratification depends on information available from standard pathology reports and archived formalin-fixed paraffin-embedded tissue (1, 2, 6, 8).

Mismatch repair deficiency has become one of the most clinically relevant molecular features in endometrial carcinoma. Immunohistochemical assessment of MLH1, PMS2, MSH2, and MSH6 is feasible in routine pathology workflows and is increasingly used to support molecular classification, Lynch syndrome screening, and therapeutic considerations in endometrial carcinoma (9–12). Deficient MMR tumors are typically considered immunogenic because of their association with microsatellite instability and increased neoantigen burden. However, the clinicopathologic implications of MMR deficiency in early-stage endometrioid endometrial carcinoma remain incompletely defined (13–15). Some dMMR tumors show immune activation and favorable biological features, whereas others present with higher grade, deeper invasion, or less favorable outcomes (6, 10, 16). These observations suggest that MMR status alone may not sufficiently characterize the immune contexture or risk profile of early-stage tumors.

The tumor immune microenvironment may partly account for this heterogeneity. Among immune cell populations, tumor-associated macrophages have attracted attention because they can participate in stromal remodeling, immune suppression, angiogenesis, and tumor invasion (17, 18). CD163 is widely used as an immunohistochemical marker of macrophage populations, although CD163 expression alone does not fully define macrophage functional polarization. Although CD163-positive macrophage infiltration has been associated with adverse features in several malignancies, its role in early-stage endometrioid endometrial carcinoma remains less well established, particularly when evaluated together with MMR status (19, 20). Most available evidence has focused on lymphocytic infiltration, PD-L1 expression, or broader molecular subtypes, whereas fewer clinicopathologic studies have examined the relationship between MMR status and CD163-positive macrophage infiltration in surgically treated early-stage disease (16, 21).

A practical challenge is that advanced immune profiling methods, such as multiplex immunofluorescence, spatial transcriptomics, or sequencing-based immune deconvolution, are not routinely available in many hospital pathology settings. In contrast, MMR and CD163 immunohistochemistry can be performed on archived surgical specimens and interpreted within a conventional histopathologic framework. The availability of both markers on archived FFPE material enables their relationship to be evaluated within a conventional clinicopathologic framework. In the present study, patients were screened from a surgically treated early-stage endometrioid endometrial carcinoma cohort between January 2020 and January 2025; after predefined exclusions, 236 patients with available tissue, clinicopathologic data, and follow-up information were analyzed. CD163-positive tumor-associated macrophage density was quantified from representative stromal and invasive-front areas, and tumors were classified into low or high infiltration groups using the cohort median cutoff.

This study primarily evaluated the association between IHC-defined MMR status and quantitative CD163-positive macrophage infiltration in early-stage endometrioid endometrial carcinoma. Secondary analyses examined associations between CD163-positive macrophage infiltration and adverse pathologic features, while the prognostic contributions of MMR status, TAM infiltration, and combined MMR–TAM phenotypes were evaluated exploratorily. By focusing on routinely accessible tissue-based markers and clinically traceable endpoints, the study sought to characterize MMR-associated CD163-positive macrophage patterns and generate hypotheses regarding their clinicopathologic and prognostic relevance.

## 2 Methods

### 2.1 Study design and patient selection

This study was designed as a single-center retrospective clinicopathologic cohort study of patients with surgically treated anatomic FIGO 2009 stage I–II endometrioid endometrial carcinoma between January 2020 and January 2025. FIGO 2009 anatomic staging was retained because stage information was abstracted from contemporaneous operative and final pathology records, whereas the complete set of granular pathologic and molecular data required for reliable retrospective FIGO 2023 reclassification was not uniformly available; p53 immunohistochemistry was not performed, and POLE sequencing, MSI testing, and MLH1 promoter methylation results were not available consistently across the cohort. Patients were identified from the institutional pathology archive and electronic medical record system, and eligibility was cross-checked against operative records, final pathology reports, and follow-up documentation. The source population consisted of 254 patients assessed for eligibility. Eligible patients were required to have histologically confirmed endometrioid endometrial carcinoma, primary surgical treatment, anatomic stage I–II disease based on the final surgical pathology report and available operative assessment, available formalin-fixed paraffin-embedded primary tumor tissue, and sufficient clinicopathologic and follow-up information for analysis.

Patients were excluded if they had non-endometrioid or mixed histology, synchronous malignancy, preoperative neoadjuvant radiotherapy or chemotherapy, documented extrauterine or nodal metastatic disease, insufficient or poor-quality paraffin tissue for immunohistochemical evaluation, missing key clinicopathologic variables, or incomplete follow-up information. After applying these criteria, 18 patients were excluded, leaving 236 patients in the final analytical cohort. No additional exclusions were made according to MMR status, CD163-positive tumor-associated macrophage infiltration, adjuvant treatment, or survival outcome. The final cohort was subsequently classified by MMR protein expression and CD163-positive macrophage infiltration to generate the four predefined MMR–TAM phenotypes used in the clinicopathologic, association, pathologic risk, and disease-free survival analyses.

### 2.2 Clinical management, pathological diagnosis, and data collection

All patients underwent primary surgical treatment consisting of total hysterectomy with bilateral salpingo-oophorectomy through laparoscopic, robotic-assisted, or open approaches according to patient condition and institutional practice. Pelvic lymph-node assessment, with or without para-aortic lymph-node sampling, was performed according to preoperative risk assessment, intraoperative findings, and the surgeon’s judgment; detailed assessment modality and node counts were not consistently documented in a form suitable for uniform retrospective subgroup analysis. Patients with pathologically confirmed nodal metastasis were not included in the final anatomic stage I–II cohort. Postoperative adjuvant treatment, including radiotherapy, chemotherapy, combined radiotherapy and chemotherapy, or observation, was recorded as part of routine clinical management and was determined by treating physicians according to postoperative pathologic risk factors and institutional practice. Adjuvant treatment was not used to define the primary exposure but was retained for descriptive and sensitivity analyses.

Clinicopathologic variables were extracted from electronic medical records, operative notes, pathology reports, and follow-up records. Demographic and clinical variables included age at surgery, menopausal status, body mass index, hypertension, diabetes mellitus, and adjuvant treatment. Pathological variables included anatomic FIGO 2009 stage, tumor grade, maximal tumor diameter, depth of myometrial invasion, lymphovascular space invasion, and lymph-node assessment status when available. Tumor grade, myometrial invasion, and LVSI were based on the final surgical pathology report and were rechecked during pathology review; for the secondary ESGO–ESTRO–ESP risk-group analysis, available H&E slides from LVSI-positive cases were additionally reviewed to distinguish focal from substantial LVSI, while the original binary LVSI variable was retained for all primary and previously specified analyses. Tumor size was recorded as the largest diameter reported in the resected specimen; when multiple measurements were available, the largest pathological measurement was used. Missing values were retained as missing and were not imputed for descriptive analyses.

### 2.3 Immunohistochemistry for MMR proteins and CD163

Immunohistochemical staining was performed on representative formalin-fixed paraffin-embedded primary tumor blocks selected from surgical specimens. For each case, 4-μm sections were prepared and stained using an automated immunohistochemistry platform (BenchMark ULTRA, Ventana Medical Systems/Roche Diagnostics, Tucson, AZ, USA). The MMR panel included MLH1 (clone M1), PMS2 (clone A16-4), MSH2 (clone G219-1129), and MSH6 (clone SP93). CD163 staining was performed using a mouse monoclonal anti-CD163 antibody (clone MRQ-26; Cell Marque/Roche Diagnostics). Antigen retrieval, endogenous peroxidase blocking, antibody incubation, OptiView DAB detection, hematoxylin counterstaining, and bluing were performed according to the manufacturer’s instructions and internal laboratory quality-control procedures. Appropriate positive and negative controls were included in each staining run.

MMR protein expression was assessed in tumor cell nuclei, with stromal cells, lymphocytes, or adjacent benign glands serving as internal positive controls. Tumors showing unequivocal retained nuclear expression of all four MMR proteins in tumor cells, with appropriate internal control staining, were classified as pMMR. Complete loss of nuclear expression of at least one MMR protein in tumor cells, in the presence of preserved nuclear staining in internal control cells, was classified as IHC-defined dMMR. Cases with equivocal or heterogeneous staining were re-reviewed on the same slide and, when necessary, on an additional representative section to avoid classification based on poor fixation, edge artifact, or tissue loss. CD163 staining was interpreted as membranous and/or cytoplasmic positivity in macrophage-like stromal cells and was subsequently quantified according to the predefined counting protocol.

### 2.4 Quantification of CD163-positive TAM infiltration and observer reproducibility

CD163-positive tumor-associated macrophage infiltration was quantified on immunostained sections using a predefined hotspot-based counting method. After scanning the entire tumor section at low magnification, areas with representative stromal macrophage infiltration were selected from the intratumoral stroma and invasive front. Necrotic areas, hemorrhage, cautery artifact, crush artifact, extensive non-specific inflammation, and poorly preserved tissue were avoided. For each case, CD163-positive macrophage-like stromal cells were manually counted in five non-overlapping high-power fields under 400× magnification using a standard light microscope (BX53, Olympus, Tokyo, Japan). With a 40× objective and a 10× eyepiece, each high-power field corresponded to approximately 0.237 mm². The mean count across the five fields was recorded as the CD163-positive TAM density and expressed as cells per high-power field. Counts from eligible intratumoral stromal and invasive-front hotspots were combined into a single case-level density rather than analyzed as separate spatial compartments.

All CD163 assessments were performed independently by two pathologists who were blinded to MMR status, clinicopathologic variables, and survival outcomes. Each pathologist independently performed hotspot selection and cell counting during blinded slide review, and discrepancies in either field selection or cell counts were resolved by joint review before assignment of the consensus case-level count. Because no clinically validated cutoff for CD163-positive TAM infiltration has been established in early-stage endometrioid endometrial carcinoma, continuous CD163-positive TAM density was emphasized as the principal quantitative measure, whereas the cohort median was retained for secondary phenotypic categorization. Tumors with CD163-positive TAM density >55 cells/HPF were classified as high TAM infiltration, whereas tumors with values ≤55 cells/HPF were classified as low TAM infiltration. The cohort median was determined from the CD163 measurements without reference to pathologic or survival outcomes. Interobserver reproducibility was assessed using agreement statistics for MMR classification and intraclass correlation for CD163-positive TAM counts, and these indices were reported to support the reliability of the immunohistochemical evaluation.

### 2.5 Definition of MMR–TAM phenotypes and study endpoints

Patients were first classified according to MMR protein expression as pMMR or dMMR and then according to CD163-positive TAM infiltration as low or high using the cohort median cutoff. These two variables were combined to generate four predefined immune-pathologic phenotypes: pMMR/low TAM, pMMR/high TAM, dMMR/low TAM, and dMMR/high TAM. This classification was retained for descriptive clinicopathologic comparisons and exploratory prognostic analyses, whereas incremental prognostic information beyond MMR status was evaluated separately using continuous CD163-positive TAM density and formal model comparison.

The principal quantitative analysis examined the association between MMR status and continuous CD163-positive TAM density, with the median-based low/high TAM categorization retained as a secondary analysis. Secondary analyses evaluated associations between CD163-positive TAM infiltration and individual adverse pathologic features, the exploratory composite pathologic endpoint, and ESGO–ESTRO–ESP clinicopathologic risk groups, whereas four-phenotype comparisons and all DFS analyses were considered exploratory. The composite adverse pathologic feature was defined as the presence of at least one of the following: grade 3 histology, deep myometrial invasion ≥50%, or LVSI positivity. This composite definition was selected to reflect routinely reported postoperative pathologic risk factors while avoiding overlap with nodal status in an early-stage cohort. For the secondary risk-group analysis, patients were classified according to the 2021 ESGO–ESTRO–ESP clinicopathologic framework for cases without molecular classification using recorded FIGO 2009 stage, tumor grade, endometrioid histology, and LVSI extent (2). Low risk comprised stage IA grade 1–2 tumors with negative or focal LVSI; intermediate risk comprised stage IB grade 1–2 tumors or stage IA grade 3 tumors with negative or focal LVSI; and high-intermediate risk comprised stage I tumors with substantial LVSI, stage IB grade 3 tumors regardless of LVSI, or stage II disease. Disease-free survival was evaluated as an exploratory prognostic endpoint according to MMR status, CD163-positive TAM infiltration, and the combined MMR–TAM phenotypes.

### 2.6 Follow-up, data completeness, and ethical considerations

Postoperative follow-up information was obtained from outpatient records, inpatient readmission records, imaging reports, pathology records for suspected recurrence, and telephone contact when necessary. The data cutoff date for follow-up was January 31, 2026. Follow-up was counted from the date of primary surgery. Disease-free survival was defined as the interval from surgery to the first documented recurrence, distant metastasis, or death from any cause. Patients without an event were censored at the date of last confirmed disease assessment or last contact. When multiple follow-up records were available within a short interval, the latest record with confirmed disease status was used to define the censoring date.

Data completeness was reviewed before statistical analysis and is summarized together with analysis-specific sample sizes in **Supplementary Table S4**. MMR status, CD163-positive TAM classification, LVSI status, and follow-up status were available for all patients included in the final cohort. Tumor size was missing in 8 of 236 patients (3.4%) and adjuvant-treatment information in 3 of 236 (1.3%); descriptive analyses used available-case denominators, and regression models involving these variables used complete cases. No statistical imputation was performed because missingness was limited, MMR status, CD163-positive TAM measurements, LVSI status, and follow-up status were complete, and missingness in tumor size or adjuvant treatment did not materially differ by MMR or TAM group. The study protocol was approved by the Medical Ethics Committee of Ganzhou Maternal and Child Health Hospital (Approval No (2025). Ethics Review Clinical No. 44). Owing to the retrospective design and the use of anonymized archival clinical and pathological data, the requirement for written informed consent was waived by the ethics committee. The study was conducted in accordance with the Declaration of Helsinki.

### 2.7 Statistical analysis

Continuous variables were assessed for normality using the Shapiro–Wilk test and are presented as mean ± standard deviation or median with interquartile range, as appropriate. Between-group comparisons were performed using Student’s t test or the Mann–Whitney U test for continuous variables. Categorical variables were compared using Pearson’s χ² test when expected cell counts were adequate and Fisher’s exact test or Monte Carlo exact testing for sparse contingency tables. CD163-positive TAM density across ESGO–ESTRO–ESP risk groups was compared using the Kruskal–Wallis test, and high versus low TAM proportions were compared using Pearson’s χ² test. Variables with missing values were summarized using available-case denominators and were not imputed. The association between MMR status and continuous CD163-positive TAM density was evaluated using median regression, whereas logistic regression was used for the secondary high-versus-low TAM analysis and for the exploratory composite adverse pathologic endpoint. Covariates were selected *a priori* to account for clinically relevant clinicopathologic heterogeneity and to limit model complexity rather than by automated variable selection; these models were interpreted as association models and not as causal models. Models evaluating MMR status in relation to CD163-positive TAM infiltration adjusted for age, FIGO stage, tumor grade, tumor size, and depth of myometrial invasion, whereas the model for the composite adverse pathologic feature adjusted for MMR status, FIGO stage, tumor size, age, and menopausal status. Disease-free survival was estimated using the Kaplan–Meier method and compared with the log-rank test. Exploratory Cox proportional hazards models evaluated MMR status alone, binary and continuous CD163-positive TAM infiltration, and the predefined dMMR/high-TAM phenotype in separate models, with adjustment for FIGO stage, tumor grade, and LVSI status. A combined model containing MMR status and continuous CD163-positive TAM density was additionally fitted, and its fit was compared with the corresponding clinical-plus-MMR model using a likelihood-ratio test. Formal MMR-by-TAM interaction terms were evaluated for the composite adverse pathologic endpoint and DFS; because only 26 DFS events occurred, penalized Cox regression was used for the DFS interaction and small-event sensitivity analyses. Because only 26 DFS events were observed, all survival models were considered exploratory and hypothesis-generating, and the number of covariates was deliberately restricted. The proportional hazards assumption was assessed using Schoenfeld residuals. Sensitivity analyses included exclusion of FIGO stage II disease for the principal MMR–TAM association, modeling CD163-positive TAM density per 10 cells/HPF for the composite pathologic endpoint, stage I-only analysis, additional adjustment of the DFS model for adjuvant therapy, exclusion of patients with follow-up shorter than 12 months, and penalized Cox regression for the exploratory DFS analysis. Multicollinearity was assessed using variance inflation factors. Statistical analyses were performed using SPSS version 26.0 (IBM Corp., Armonk, NY, USA) and R version 4.3.3 (R Foundation for Statistical Computing, Vienna, Austria). All tests were two-sided, and P values <0.05 were considered statistically significant. No formal multiplicity adjustment was applied; therefore, secondary analyses, four-phenotype comparisons, interaction analyses, sensitivity analyses, and all DFS analyses were interpreted as exploratory.

## 3 Results

### 3.1 Cohort characteristics and immunopathologic classification

Among 254 patients assessed for eligibility, 18 were excluded because of non-endometrioid or mixed histology, neoadjuvant therapy before surgery, inadequate paraffin tissue for immunohistochemistry, missing key clinicopathologic data, or incomplete follow-up information. The final analytical cohort included 236 patients with surgically treated early-stage endometrioid endometrial carcinoma (**Figure 1**). Overall, the median age was 57 years, and 151 patients (64.0%) were postmenopausal. Most tumors were FIGO stage I, including 130 stage IA and 62 stage IB cases, while 44 patients (18.6%) had stage II disease. Grade 3 histology was present in 55 patients (23.3%), deep myometrial invasion in 94 (39.8%), LVSI in 41 (17.4%), and a composite adverse pathologic feature in 89 (37.7%) (**Table 1**).

**Figure 1:**
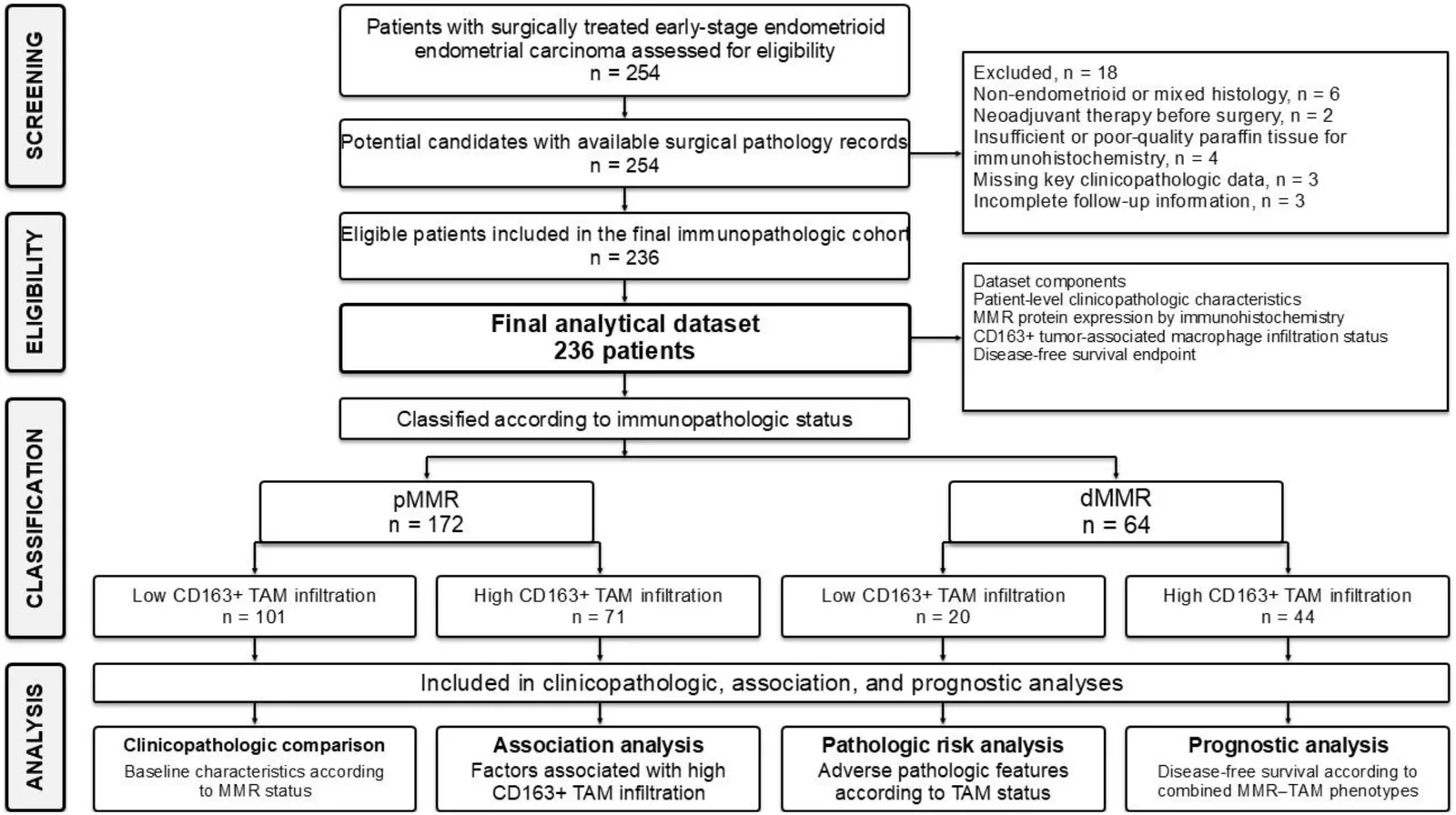
Patient selection, immunopathologic classification, and analytical framework. A total of 254 patients with surgically treated early-stage endometrioid endometrial carcinoma were assessed for eligibility. After exclusion of 18 patients because of ineligible histology, neoadjuvant therapy, inadequate paraffin tissue, missing key clinicopathologic data, or incomplete follow-up information, 236 patients were included in the final analytical cohort. Tumors were classified as pMMR (n=172) or IHC-defined dMMR (n=64) and subsequently categorized as low or high CD163-positive TAM infiltration using the cohort-median threshold of 55 cells/HPF, generating four predefined phenotypes: pMMR/low TAM (n=101), pMMR/high TAM (n=71), dMMR/low TAM (n=20), and dMMR/high TAM (n=44). The principal analysis evaluated the association between MMR status and CD163-positive TAM infiltration. Associations with adverse pathologic features were evaluated as secondary analyses, whereas combined MMR–TAM phenotype and disease-free survival analyses were considered exploratory. MMR, mismatch repair; pMMR, proficient mismatch repair; dMMR, deficient mismatch repair; TAM, tumor-associated macrophage; HPF, high-power field.

**Table 1:** Baseline clinicopathologic characteristics according to MMR status.

Variable	pMMR (n = 172)	dMMR (n = 64)	Test statistic	P value
Age, years	56 (49–64)	59 (52–67)	Z = 1.73	0.084
Postmenopausal status	107 (62.2)	44 (68.8)	χ² = 0.87	0.352
Body mass index, kg/m²	27.0 ± 4.8	27.6 ± 4.7	t = 0.87	0.388
Hypertension	58 (33.7)	25 (39.1)	χ² = 0.58	0.445
Diabetes mellitus	31 (18.0)	12 (18.8)	χ² = 0.02	0.898
Tumor size ≥3 cm, n/N (%)	75/167 (44.9)	32/61 (52.5)	χ² = 1.02	0.312
FIGO stage IA	99 (57.6)	31 (48.4)		
FIGO stage IB	42 (24.4)	20 (31.3)	χ² = 1.66	0.435
FIGO stage II	31 (18.0)	13 (20.3)		
Grade 1	70 (40.7)	14 (21.9)		
Grade 2	68 (39.5)	29 (45.3)	χ² = 8.43	0.015
Grade 3	34 (19.8)	21 (32.8)		
Deep myometrial invasion ≥50%	60 (34.9)	34 (53.1)	χ² = 6.48	0.011
LVSI positive	25 (14.5)	16 (25.0)	χ² = 3.56	0.059
Composite adverse pathologic feature	61 (35.5)	28 (43.8)	χ² = 1.36	0.243
High CD163+ TAM infiltration	71 (41.3)	44 (68.8)	χ² = 14.09	<0.001
Adjuvant radiotherapy, n/N (%)	46/170 (27.1)	20/63 (31.7)	χ² = 0.50	0.481
Adjuvant chemotherapy, n/N (%)	25/170 (14.7)	14/63 (22.2)	χ² = 1.86	0.172
Follow-up, months	48 (36–58)	49 (37–59)	Z = 0.46	0.642

Values are n (%), n/N (%), median (IQR), or mean ± SD. Percentages for variables with missing data were calculated using available-case denominators. Composite adverse pathologic feature was defined as grade 3 histology, deep myometrial invasion ≥50%, or LVSI positivity.

MMR protein expression was retained in 172 tumors (72.9%), whereas 64 tumors (27.1%) were classified as dMMR. The most common loss pattern was MLH1/PMS2 loss (44/64, 68.8%), followed by MSH2/MSH6 loss (9/64, 14.1%), isolated MSH6 loss (7/64, 10.9%), and isolated PMS2 loss (4/64, 6.3%) (**Table 2**). The median CD163-positive TAM density was 55 cells/HPF, and 115 tumors (48.7%) were classified as high TAM infiltration using the cohort median cutoff. Representative histopathological and immunohistochemical findings of MMR status and CD163-positive TAM infiltration are shown in **Figure 2**. Combining MMR status and TAM infiltration generated four phenotypes: pMMR/low TAM (n = 101), pMMR/high TAM (n = 71), dMMR/low TAM (n = 20), and dMMR/high TAM (n = 44), which were used for subsequent clinicopathologic and survival analyses. Data completeness, interobserver agreement, multicollinearity assessment, and proportional hazards diagnostics are summarized in **Supplementary Table S4**.

**Table 2:** Immunohistochemical profiles and CD163+ TAM quantification.

Variable	Overall (n = 236)	pMMR (n = 172)	dMMR (n = 64)	Test statistic	P value
Retained expression of all four MMR proteins	172 (72.9)	172 (100.0)	0 (0.0)	—	—
Any MMR protein loss	64 (27.1)	0 (0.0)	64 (100.0)	—	—
MLH1/PMS2 loss	44 (18.6)	0 (0.0)	44 (68.8)	—	—
MSH2/MSH6 loss	9 (3.8)	0 (0.0)	9 (14.1)	—	—
Isolated MSH6 loss	7 (3.0)	0 (0.0)	7 (10.9)	—	—
Isolated PMS2 loss	4 (1.7)	0 (0.0)	4 (6.3)	—	—
CD163+ TAM density, cells/HPF	55 (31–84)	47 (26–74)	76 (48–98)	Z = 4.21	<0.001
High CD163+ TAM infiltration	115 (48.7)	71 (41.3)	44 (68.8)	χ² = 14.09	<0.001
Low CD163+ TAM infiltration	121 (51.3)	101 (58.7)	20 (31.3)	χ² = 14.09	<0.001
Interobserver agreement for MMR status	κ = 0.94 (0.89–0.98)	—	—	—	—
Interobserver agreement for CD163+ TAM count	ICC = 0.88 (0.84–0.91)	—	—	—	—

Values are n (%), median (IQR), or agreement coefficient with 95% CI. High CD163+ TAM infiltration was defined as CD163+ TAM density >55 cells/HPF, whereas values ≤55 cells/HPF were classified as low infiltration. MMR, mismatch repair; TAM, tumor-associated macrophage; HPF, high-power field; ICC, intraclass correlation coefficient.

**Figure 2:**
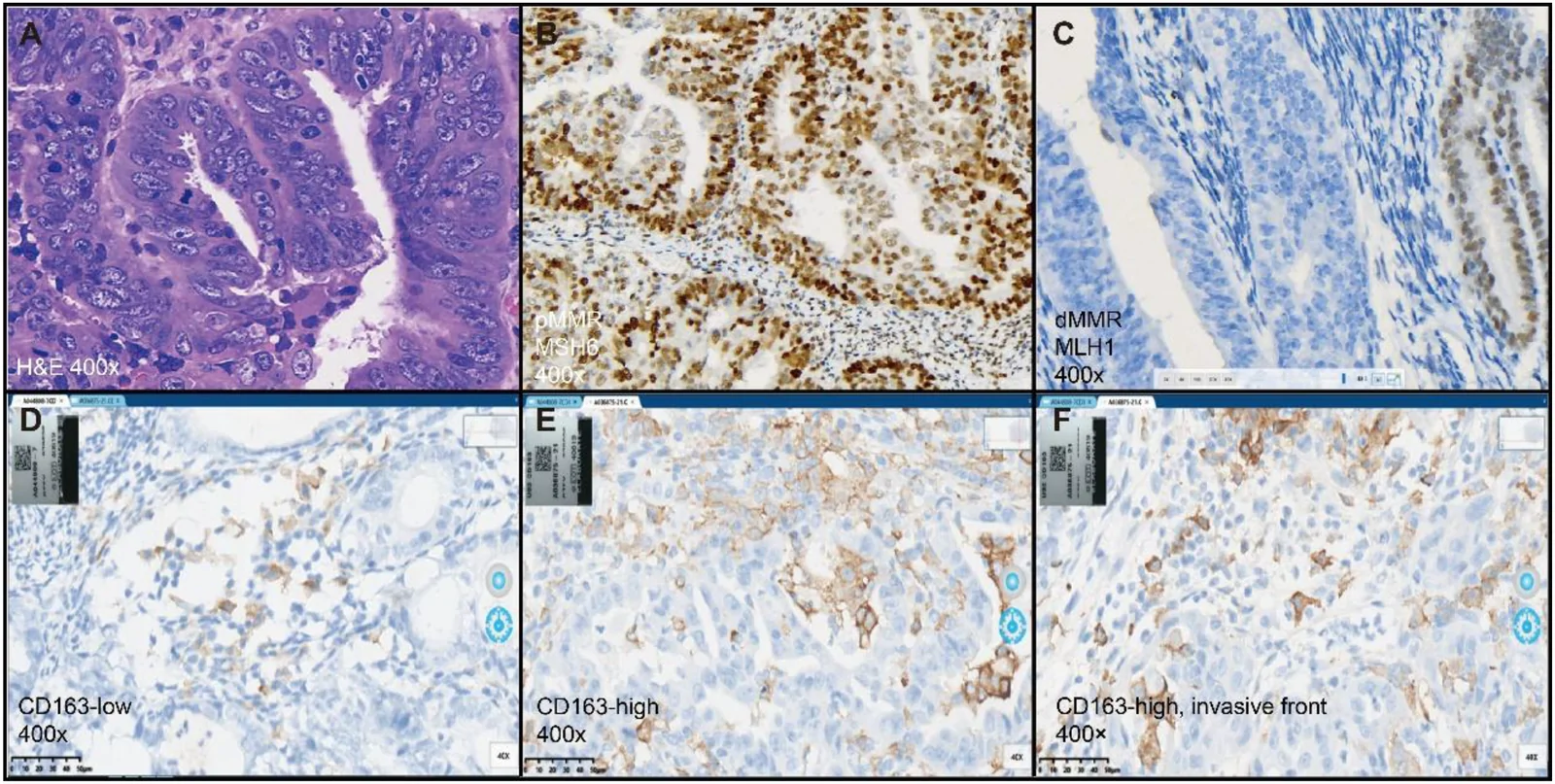
Representative histopathological and immunohistochemical findings of MMR status and CD163-positive TAM infiltration in early-stage endometrioid endometrial carcinoma. **(A)** Representative H&E image. **(B)** Representative pMMR tumor showing retained nuclear expression of MSH6. **(C)** Representative dMMR tumor showing loss of MLH1 expression in tumor cells, with retained staining in non-neoplastic cells serving as an internal positive control. **(D)** Representative low CD163-positive TAM infiltration. **(E)** Representative high CD163-positive TAM infiltration. **(F)** Representative high CD163-positive TAM infiltration at the invasive front. Original magnification, ×400 for all panels.

### 3.2 MMR deficiency was associated with increased CD163-positive TAM infiltration

In the principal quantitative analysis, CD163-positive TAM density was higher in dMMR tumors than in pMMR tumors. Median CD163-positive TAM density was 76 cells/HPF (IQR, 48–98) in dMMR tumors and 47 cells/HPF (IQR, 26–74) in pMMR tumors (P<0.001) (**Figure 3A**; **Table 2**). After adjustment for age, FIGO stage, tumor grade, tumor size, and depth of myometrial invasion, dMMR remained associated with higher continuous CD163-positive TAM density, with an adjusted median difference of 22 cells/HPF (95% CI, 10–34; P<0.001) (**Table 3**). In the secondary median-based analysis, high TAM infiltration was observed in 44 of 64 dMMR tumors (68.8%) and 71 of 172 pMMR tumors (41.3%; P<0.001) (**Figure 3B**; **Table 2**), supporting concordant associations across continuous and binary representations of CD163-positive TAM infiltration.

**Figure 3:**
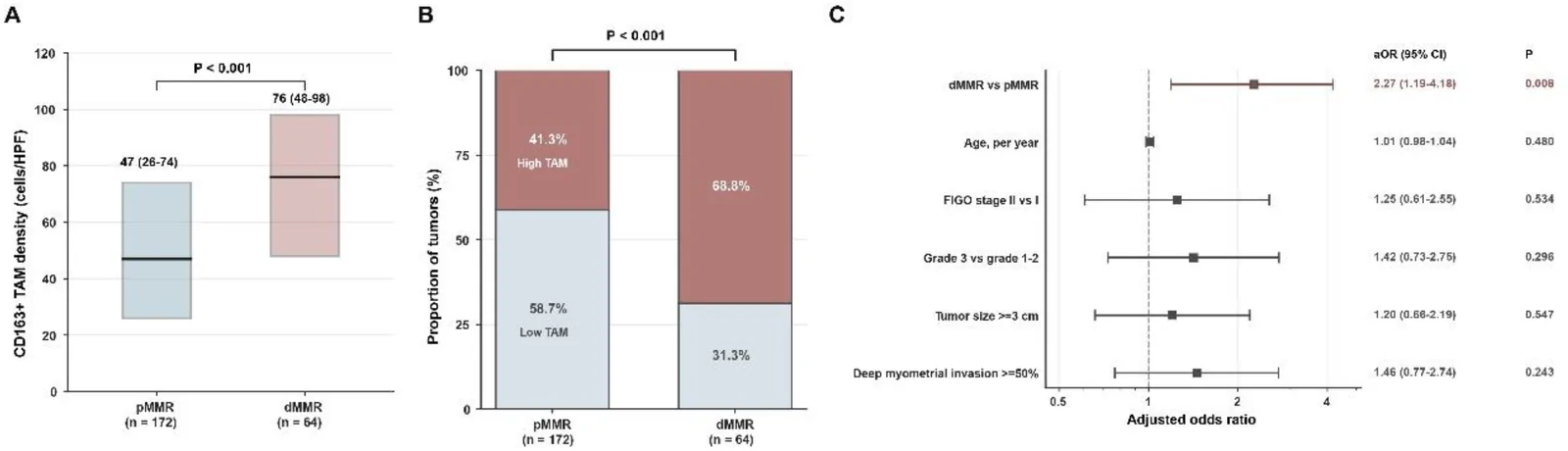
Association between mismatch repair status and CD163-positive tumor-associated macrophage infiltration. **(A)** CD163-positive TAM density according to MMR status. Boxes indicate the interquartile range and horizontal lines indicate the median. Median CD163-positive TAM density was 47 cells/HPF (IQR, 26–74) in pMMR tumors and 76 cells/HPF (IQR, 48–98) in dMMR tumors (P<0.001). **(B)** Distribution of low and high CD163-positive TAM infiltration according to MMR status. High infiltration was observed in 71 of 172 pMMR tumors (41.3%) and 44 of 64 dMMR tumors (68.8%; P<0.001). Low and high infiltration were defined using the cohort-median threshold of 55 cells/HPF; this categorization was used for secondary phenotypic analyses and does not represent a clinically validated cutoff. **(C)** Multivariable logistic regression analysis of factors associated with high CD163-positive TAM infiltration. Estimates were adjusted for age, FIGO stage, tumor grade, tumor size, and depth of myometrial invasion. Squares indicate adjusted odds ratios and horizontal capped lines indicate 95% confidence intervals. MMR, mismatch repair; pMMR, proficient mismatch repair; dMMR, deficient mismatch repair; TAM, tumor-associated macrophage; HPF, high-power field; aOR, adjusted odds ratio; CI, confidence interval.

**Table 3:** Association between mismatch repair status and CD163-positive tumor-associated macrophage infiltration.

Outcome or analysis	pMMR (n = 172)	dMMR (n = 64)	Unadjusted effect estimate	P value	Adjusted effect estimate (95% CI)	P value	Analysis n
CD163+ TAM density, cells/HPF	47 (26–74)	76 (48–98)	Median difference, 29 cells/HPF	<0.001	Adjusted median difference, 22 cells/HPF (10–34)	<0.001	228
High CD163+ TAM infiltration	71 (41.3)	44 (68.8)	OR 3.13 (1.70–5.76)	<0.001	OR 2.27 (1.19–4.18)	0.008	228

Values are median (IQR) or n (%). The continuous CD163-positive TAM density analysis was emphasized as the principal quantitative analysis because no externally validated clinical threshold for CD163-positive macrophage infiltration exists in early-stage endometrioid endometrial carcinoma. The adjusted continuous analysis used median regression, and the adjusted binary analysis used logistic regression. Both models included age, FIGO stage, tumor grade, tumor size, and depth of myometrial invasion. High TAM infiltration was defined as >55 cells/HPF and low TAM infiltration as ≤55 cells/HPF. The 55-cells/HPF threshold was derived from the cohort median and was used for secondary phenotypic analyses rather than as a clinically validated cutoff. Complete-case analysis was used for multivariable models. MMR, mismatch repair; pMMR, proficient mismatch repair; dMMR, deficient mismatch repair; TAM, tumor-associated macrophage; HPF, high-power field; OR, odds ratio; CI, confidence interval.

In logistic regression analysis, dMMR status was associated with high CD163-positive TAM infiltration in the unadjusted model (OR, 3.13; 95% CI, 1.70–5.76; P < 0.001). After adjustment for age, FIGO stage, tumor grade, tumor size, and deep myometrial invasion, IHC-defined dMMR remained associated with high CD163-positive TAM infiltration (adjusted OR, 2.27; 95% CI, 1.19–4.18; P = 0.008) (**Figure 3C**; **Table 3**).

### 3.3 CD163-positive TAM infiltration was associated with adverse pathologic features

Tumors with high CD163-positive TAM infiltration showed a higher frequency of several adverse postoperative pathologic features than tumors with low infiltration (**Figure 4A**; **Table 4**). Grade 3 histology was observed in 35 of 115 high-TAM tumors (30.4%) compared with 20 of 121 low-TAM tumors (16.5%; χ² = 6.38, P = 0.012). Deep myometrial invasion ≥50% was also more frequent in the high-TAM group (58/115, 50.4%) than in the low-TAM group (36/121, 29.8%; χ² = 10.52, P = 0.001), as was LVSI positivity (28/115, 24.3% vs 13/121, 10.7%; χ² = 7.60, P = 0.006). The exploratory composite adverse pathologic feature was present in 56 of 115 high-TAM tumors (48.7%) and 33 of 121 low-TAM tumors (27.3%; P = 0.001).

**Figure 4:**
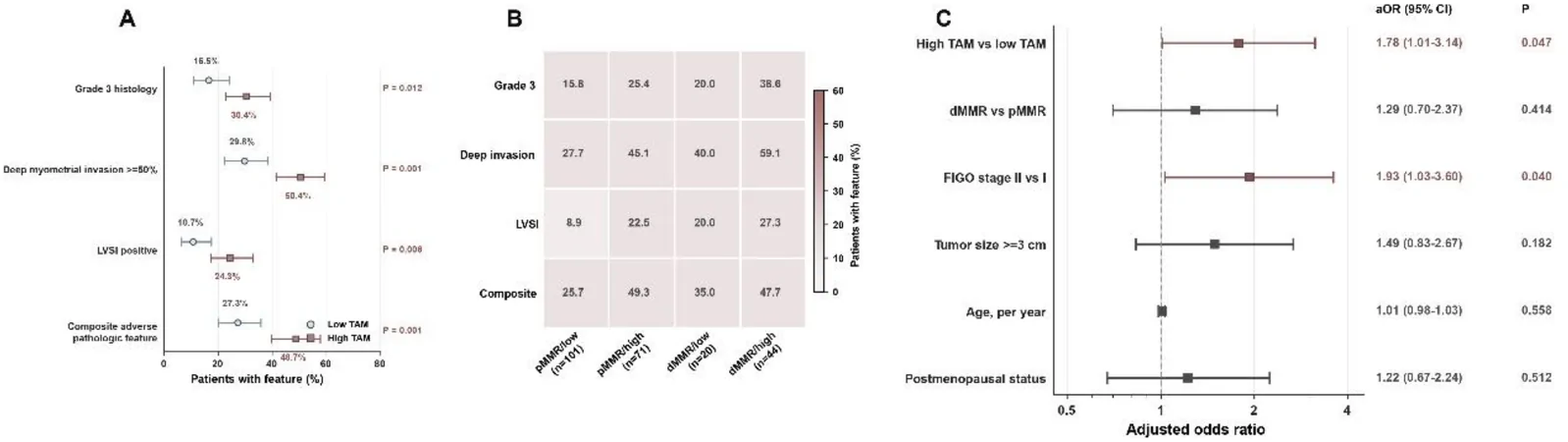
Associations between CD163-positive TAM infiltration and adverse pathologic features. **(A)** Frequencies of grade 3 histology, deep myometrial invasion ≥50%, LVSI positivity, and the composite adverse pathologic feature according to low versus high CD163-positive TAM infiltration. Points indicate observed proportions and horizontal capped lines indicate 95% confidence intervals. **(B)** Distribution of adverse pathologic features across the four predefined MMR–TAM phenotypes. Values represent percentages within each phenotype. The four-group comparison is descriptive and should not be interpreted as demonstrating a monotonic or graded pattern across phenotypes. **(C)** Multivariable logistic regression analysis of factors associated with the composite adverse pathologic feature. The model included TAM status, MMR status, FIGO stage, tumor size, age, and menopausal status. Squares indicate adjusted odds ratios and horizontal capped lines indicate 95% confidence intervals. These findings represent clinicopathologic associations and do not establish a causal effect of macrophage infiltration on tumor grade, myometrial invasion, or LVSI. MMR, mismatch repair; TAM, tumor-associated macrophage; LVSI, lymphovascular space invasion; aOR, adjusted odds ratio; CI, confidence interval.

**Table 4:** Associations between CD163-positive TAM infiltration and adverse pathologic features.

Pathologic outcome	Low TAM (n = 121)	High TAM (n = 115)	Unadjusted OR (95% CI)	P value	Adjusted OR (95% CI)	P value
Grade 3 histology	20 (16.5)	35 (30.4)	2.21 (1.19–4.12)	0.012	—	—
Deep myometrial invasion ≥50%	36 (29.8)	58 (50.4)	2.40 (1.41–4.10)	0.001	—	—
LVSI positive	13 (10.7)	28 (24.3)	2.67 (1.31–5.47)	0.006	—	—
Composite adverse pathologic feature	33 (27.3)	56 (48.7)	2.53 (1.47–4.35)	0.001	1.78 (1.01–3.14)	0.047

Values are n (%). The composite adverse pathologic feature was defined as the presence of at least one of the following: grade 3 histology, deep myometrial invasion ≥50%, or LVSI positivity. Individual components are presented separately because the composite endpoint does not represent a single homogeneous pathologic entity. To avoid redundant multivariable analyses and excessive multiple testing, only the predefined exploratory composite endpoint was evaluated in an adjusted logistic model. The adjusted model included MMR status, FIGO stage, tumor size, age, and menopausal status and used complete cases (n = 228). Associations are clinicopathologic associations and should not be interpreted as demonstrating that CD163-positive macrophage infiltration causally produces tumor grade, myometrial invasion, or LVSI. LVSI, lymphovascular space invasion; TAM, tumor-associated macrophage; OR, odds ratio; CI, confidence interval.

Postoperative treatment differed according to TAM group, consistent with treatment allocation based on postoperative clinicopathologic risk factors. Adjuvant chemotherapy was recorded in 25 of 113 high-TAM patients (22.1%) and 14 of 120 low-TAM patients (11.7%; P = 0.033), whereas adjuvant radiotherapy was recorded in 38 of 113 (33.6%) and 28 of 120 (23.3%), respectively (P = 0.081) (**Supplementary Table S1**). In the multivariable logistic model for the composite adverse pathologic feature, high TAM infiltration remained associated with increased odds after adjustment for MMR status, FIGO stage, tumor size, age, and menopausal status (adjusted OR, 1.78; 95% CI, 1.01–3.14; P = 0.047) (**Figure 4C**). FIGO stage II also retained an association in this model (adjusted OR, 1.93; 95% CI, 1.03–3.60; P = 0.040). CD163-positive TAM infiltration also differed across the ESGO–ESTRO–ESP clinicopathologic risk groups: median density was 46 cells/HPF (IQR, 28–72), 55 cells/HPF (IQR, 33–80), and 63 cells/HPF (IQR, 40–91) in the low-, intermediate-, and high-intermediate-risk groups, respectively (global P = 0.016), while high TAM infiltration occurred in 38/98 (38.8%), 32/66 (48.5%), and 45/72 (62.5%) patients, respectively (global P = 0.009) (**Supplementary Table S5**).

### 3.4 Adverse pathologic features differed across combined MMR–TAM phenotypes

When MMR status and CD163-positive TAM infiltration were combined, the four immune-pathologic phenotypes showed different distributions of adverse pathologic features (**Figure 4B**; **Supplementary Table S2**). The four phenotypes showed heterogeneous and non-monotonic distributions of adverse pathologic features, with the pMMR/low-TAM group generally showing lower absolute frequencies and the remaining groups showing variable patterns (**Figure 4B**; **Supplementary Table S2**). Grade 3 histology was present in 16 of 101 pMMR/low TAM tumors (15.8%), 18 of 71 pMMR/high TAM tumors (25.4%), 4 of 20 dMMR/low TAM tumors (20.0%), and 17 of 44 dMMR/high TAM tumors (38.6%; P = 0.026). A similar distribution was observed for deep myometrial invasion ≥50%, which was recorded in 27.7%, 45.1%, 40.0%, and 59.1% of the four groups, respectively (P = 0.003).

LVSI positivity was also least frequent in the pMMR/low TAM group (9/101, 8.9%) and more common in the pMMR/high TAM, dMMR/low TAM, and dMMR/high TAM groups (22.5%, 20.0%, and 27.3%, respectively; P = 0.024). The composite adverse pathologic feature was present in 26 of 101 pMMR/low TAM tumors (25.7%), compared with 35 of 71 pMMR/high TAM tumors (49.3%), 7 of 20 dMMR/low TAM tumors (35.0%), and 21 of 44 dMMR/high TAM tumors (47.7%; P = 0.007). Global four-group comparisons were considered descriptive and exploratory, and no specific pairwise differences or monotonic ordering were inferred from these P values (**Supplementary Table S2**).

### 3.5 Exploratory disease-free survival analyses according to MMR status and CD163-positive TAM infiltration

During follow-up, 26 DFS events were recorded: 14 among 64 patients with dMMR tumors and 12 among 172 patients with pMMR tumors. In the exploratory single-marker Cox analysis, dMMR was associated with DFS after adjustment for FIGO stage, tumor grade, and LVSI (adjusted HR, 2.76; 95% CI, 1.25–6.10; P = 0.012) (**Figures 5A, C**; **Table 5**). In contrast, high versus low TAM infiltration was not significantly associated with DFS after adjustment (adjusted HR, 1.55; 95% CI, 0.68–3.53; P = 0.297), and continuous CD163-positive TAM density likewise showed no statistically significant adjusted association per 10 cells/HPF (adjusted HR, 1.04; 95% CI, 0.97–1.12; P = 0.281) (**Figures 5B, C**; **Table 5**). In the model containing both MMR status and continuous CD163-positive TAM density, dMMR remained associated with DFS (adjusted HR, 2.65; 95% CI, 1.20–5.86; P = 0.016), whereas continuous CD163-positive TAM density did not (adjusted HR, 1.04; 95% CI, 0.97–1.12; P = 0.279). Addition of continuous CD163-positive TAM density did not significantly improve model fit beyond the corresponding clinical-plus-MMR model (likelihood-ratio χ²=1.17; P = 0.279) (**Table 5**).

**Figure 5:**
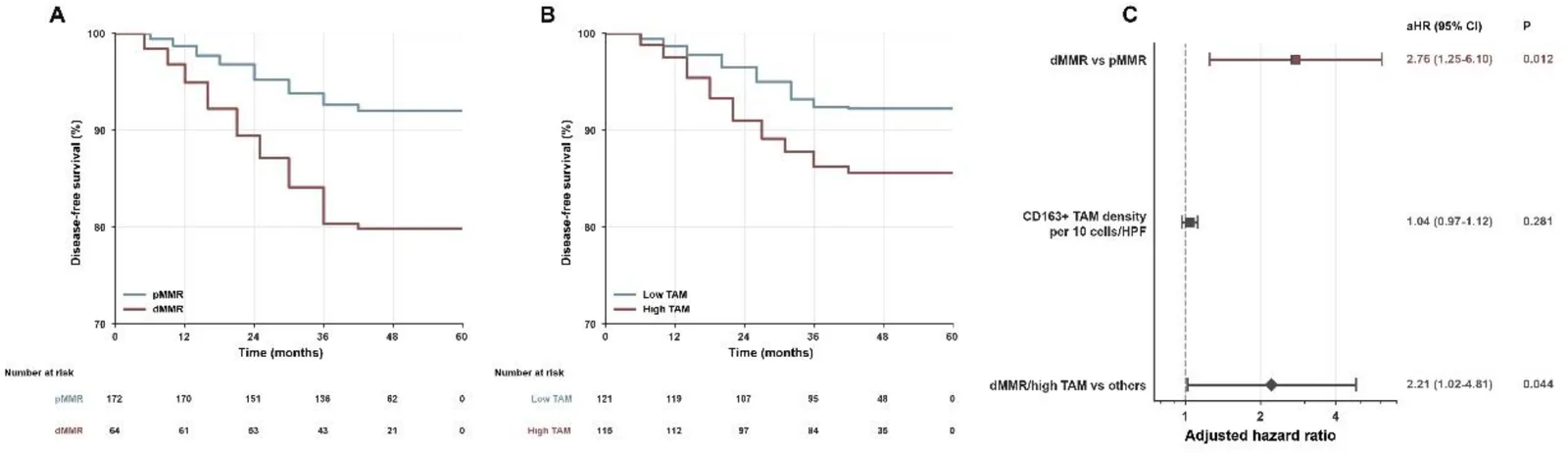
Exploratory disease-free survival analyses according to MMR status and CD163-positive TAM infiltration. **(A)** Kaplan–Meier estimates of disease-free survival according to MMR status. The analysis included 172 patients with pMMR tumors and 64 with dMMR tumors, with 12 and 14 DFS events, respectively. **(B)** Kaplan–Meier estimates of disease-free survival according to low versus high CD163-positive TAM infiltration. The analysis included 121 patients with low TAM infiltration and 115 with high TAM infiltration, with 9 and 17 DFS events, respectively. Numbers at risk are shown below the curves. **(C)** Selected adjusted hazard-ratio estimates from separate exploratory Cox regression models. The dMMR-versus-pMMR estimate was derived from the MMR single-marker model, the continuous CD163-positive TAM estimate from the continuous-TAM model, and the dMMR/high-TAM estimate from the predefined combined-phenotype model. Each model was adjusted for FIGO stage, tumor grade, and LVSI status. Squares or diamonds indicate adjusted hazard ratios and horizontal capped lines indicate 95% confidence intervals. Because only 26 DFS events occurred, all survival analyses were considered exploratory and hypothesis-generating. DFS, disease-free survival; MMR, mismatch repair; pMMR, proficient mismatch repair; dMMR, deficient mismatch repair; TAM, tumor-associated macrophage; HPF, high-power field; LVSI, lymphovascular space invasion; aHR, adjusted hazard ratio; CI, confidence interval.

**Table 5:** Exploratory Cox regression analyses of disease-free survival.

Analysis	Exposure or comparison	DFS events/total	Unadjusted HR (95% CI)	P value	Adjusted HR or test estimate (95% CI)	P value
Single-marker MMR analysis	dMMR vs pMMR	14/64 vs 12/172	3.30 (1.54–7.09)	0.002	2.76 (1.25–6.10)	0.012
Single-marker binary TAM analysis	High TAM vs low TAM	17/115 vs 9/121	2.03 (0.91–4.53)	0.084	1.55 (0.68–3.53)	0.297
Single-marker continuous TAM analysis	CD163+ TAM density, per 10 cells/HPF	26/236	1.06 (0.99–1.13)	0.089	1.04 (0.97–1.12)	0.281
Combined MMR + continuous TAM model	dMMR vs pMMR	26/236	—	—	2.65 (1.20–5.86)	0.016
Combined MMR + continuous TAM model	CD163+ TAM density, per 10 cells/HPF	26/236	—	—	1.04 (0.97–1.12)	0.279
Incremental model comparison	Clinical + MMR + CD163 vs clinical + MMR	26/236	—	—	Likelihood-ratio χ² = 1.17	0.279
Exploratory combined phenotype	dMMR/high TAM vs all other phenotypes	11/44 vs 15/192	2.48 (1.14–5.37)	0.022	2.21 (1.02–4.81)	0.044

All DFS analyses were considered exploratory because only 26 DFS events occurred. Conventional adjusted Cox models included FIGO stage, tumor grade, and LVSI status together with the principal exposure shown in the table. The combined MMR + continuous TAM model included both MMR status and CD163-positive TAM density in addition to the same clinical covariates. Addition of continuous CD163-positive TAM density did not significantly improve model fit beyond the clinical + MMR model according to the likelihood-ratio test. The dMMR/high-TAM phenotype analysis was retained as an exploratory phenotypic analysis and should not be interpreted as demonstrating statistical interaction or validated incremental prognostic value beyond MMR status. Small-event, interaction, treatment-adjusted, and follow-up sensitivity analyses are reported in **Supplementary Table S3**. DFS, disease-free survival; HR, hazard ratio; CI, confidence interval; MMR, mismatch repair; dMMR, deficient mismatch repair; pMMR, proficient mismatch repair; TAM, tumor-associated macrophage; HPF, high-power field; LVSI, lymphovascular space invasion.

The predefined dMMR/high-TAM phenotype was retained as an exploratory analysis: 11 of 44 patients experienced a DFS event compared with 15 of 192 patients in the remaining phenotypes, and estimated 3-year DFS was 78.4% versus 92.3% (log-rank P = 0.018) (**Supplementary Figure S1**). In the conventional exploratory Cox model, the dMMR/high-TAM phenotype had an adjusted HR of 2.21 (95% CI, 1.02–4.81; P = 0.044); however, the estimate was attenuated after additional adjustment for adjuvant therapy (HR, 2.04; 95% CI, 0.93–4.52; P = 0.075) and in penalized Cox regression (HR, 2.14; 95% CI, 0.99–4.62; P = 0.053) (**Table 5**; **Supplementary Table S3**). Formal interaction analyses did not support effect modification between MMR status and high TAM infiltration for either the composite adverse pathologic endpoint (interaction OR, 0.60; 95% CI, 0.17–2.15; P = 0.437) or DFS (interaction HR, 1.18; 95% CI, 0.34–4.10; P = 0.793) (**Supplementary Table S3**). Additional stage-restricted, continuous-density, treatment-adjusted, and follow-up sensitivity analyses are summarized in **Supplementary Table S3**.

## 4 Discussion

In this single-center retrospective clinicopathologic cohort of 236 patients with surgically treated early-stage endometrioid endometrial carcinoma, the most robust finding was an association between IHC-defined dMMR and increased CD163-positive macrophage infiltration (6, 21). High CD163-positive TAM infiltration was observed in 44 of 64 dMMR tumors, compared with 71 of 172 pMMR tumors, and this association persisted after adjustment for age, FIGO stage, tumor grade, tumor size, and deep myometrial invasion. CD163-positive TAM infiltration was also linked to a higher burden of adverse pathologic features, including grade 3 histology, deep myometrial invasion, LVSI, and the composite adverse pathologic feature (17, 20). DFS analyses were exploratory: MMR status showed a stronger association with DFS than TAM infiltration, whereas formal interaction testing and model comparison did not demonstrate statistically supported incremental prognostic information from CD163-positive TAM infiltration beyond MMR status.

The association between dMMR and CD163-positive TAM infiltration adds a more nuanced view to the conventional understanding of dMMR tumors as immunologically active. In the present cohort, dMMR was not only associated with higher categorical TAM infiltration but also with higher quantitative CD163-positive macrophage density (10, 20). This finding supports a quantitative association between dMMR and CD163-positive macrophage density but does not establish macrophage functional polarization or the overall composition of the tumor immune microenvironment. The association between dMMR and CD163-positive TAM infiltration persisted after adjustment for the prespecified clinicopathologic variables, although the observational design and the role of some pathologic variables in tumor evolution preclude causal interpretation (18, 22). However, this study cannot determine whether MMR deficiency promotes macrophage recruitment, whether macrophage infiltration reflects a broader inflammatory response, or whether both are consequences of unmeasured tumor biology (21).

The pathological findings provide a clinically traceable context for the immune findings. High TAM infiltration was associated with higher rates of grade 3 histology, deep myometrial invasion, and LVSI, all of which are routinely reported postoperative risk factors in early-stage endometrioid endometrial carcinoma (15, 23, 24). The composite adverse pathologic feature was present in 56 of 115 high-TAM tumors compared with 33 of 121 low-TAM tumors, and the association remained statistically significant after adjustment, although the confidence interval was close to the null. These findings support an association between CD163-positive TAM infiltration and a greater frequency of adverse pathologic characteristics in this cohort, without establishing the direction of that relationship. The secondary ESGO–ESTRO–ESP risk-group analysis was directionally consistent with these clinicopathologic associations, with greater CD163-positive TAM density and a higher proportion of high TAM infiltration in higher-risk groups; this analysis was descriptive and does not establish independent prognostic value. The higher frequency of adjuvant chemotherapy among high-TAM patients is compatible with postoperative risk-based treatment allocation and introduces potential confounding by indication into the DFS analyses (5, 25, 26).

The combined MMR–TAM classification was retained as a descriptive framework for examining clinicopathologic heterogeneity rather than as an established prognostic classification. The pMMR/low TAM group had the lowest absolute frequency of several adverse pathologic features, whereas the dMMR/high TAM group showed higher rates of grade 3 histology, deep myometrial invasion, LVSI, and DFS events. The non-monotonic distribution of adverse pathologic features across the four phenotypes, together with the non-significant interaction tests, argues against interpreting the combined groups as evidence of biological synergy or a graded risk hierarchy. The four-group MMR–TAM classification therefore serves primarily as a descriptive tissue-based grouping in this cohort and should not be considered a substitute for established staging or molecular classification systems. The DFS analyses remained exploratory and were limited by only 26 events. In the single-marker Cox model, dMMR was associated with DFS with an adjusted HR of 2.76 (95% CI, 1.25–6.10), a relatively large effect estimate for an early-stage cohort; its magnitude should be interpreted cautiously because the confidence interval was wide and unmeasured molecular heterogeneity, including unrecognized p53-abnormal or POLE-mutated tumors, may have influenced the estimate. The dMMR/high TAM group had 11 DFS events among 44 patients, compared with 15 events among 192 patients in the remaining phenotypes. In the Cox model, the adjusted HR for the dMMR/high TAM phenotype was 2.21, with a lower confidence limit close to 1.00. After additional adjustment for adjuvant therapy, the association between the dMMR/high TAM phenotype and DFS was attenuated and no longer met conventional statistical significance, while remaining directionally consistent (27). Taken together, the survival results do not establish incremental prognostic value of CD163-positive TAM infiltration beyond MMR status and should be regarded as hypothesis-generating. Larger cohorts with more events are needed before this phenotype can be considered for clinical risk prediction (28, 29).

This study has several limitations. First, its retrospective single-center design may introduce selection bias, particularly because only patients with adequate archival tissue and complete follow-up were included. Second, CD163 was used as a single macrophage marker and therefore cannot define a homogeneous M2 phenotype, macrophage functional state, or the broader immune-cell composition and spatial context of the tumor microenvironment. Third, the 55-cells/HPF threshold was derived from the cohort median and was used only for secondary phenotypic analyses; it should not be interpreted as a clinically validated or externally transferable threshold. Fourth, only 26 DFS events occurred, limiting multivariable model complexity and producing imprecise estimates; the attenuation observed after adjustment for adjuvant therapy and in penalized Cox regression further supports cautious interpretation. Fifth, the study assessed four-protein MMR immunohistochemistry, but p53 immunohistochemistry was not performed, and POLE sequencing, MSI testing, and MLH1 promoter methylation results were not available consistently across the cohort; consequently, complete contemporary molecular classification was unavailable, and the observed associations may be affected by unmeasured molecular heterogeneity (30). Lymph-node assessment was not uniform, and the available retrospective records did not permit reliable reconstruction of all elements required for FIGO 2023 staging; the cohort was therefore analyzed using the recorded anatomic FIGO 2009 framework. Finally, postoperative treatment was assigned according to real-world clinical judgment and may have confounded the association between immune-pathologic phenotype and DFS. Although missingness was limited, the use of complete-case regression for variables with missing observations remains a potential source of residual selection bias, and the absence of external validation further limits clinical generalizability.

## 5 Conclusion

In early-stage endometrioid endometrial carcinoma, IHC-defined MMR deficiency was associated with increased quantitative and categorical CD163-positive TAM infiltration, and higher TAM infiltration was associated with a greater frequency of adverse pathologic features. DFS analyses were exploratory; MMR status showed a stronger association with DFS than TAM infiltration, and neither formal interaction testing nor model comparison demonstrated clear incremental prognostic information from TAM infiltration beyond MMR status. These findings support further investigation of the relationship between MMR status and CD163-positive macrophage infiltration, with larger externally validated cohorts required to determine whether these observations have independent prognostic relevance.

## Data Availability

The raw data supporting the conclusions of this article will be made available by the authors, without undue reservation.

## Supplementary material

Supplementary Table 5 CD163-positive TAM infiltration across ESGO–ESTRO–ESP clinicopathologic risk groups. Risk groups were assigned according to the 2021 ESGO–ESTRO–ESP clinicopathologic framework for patients with unknown molecular classification. Low risk included FIGO 2009 stage IA endometrioid carcinoma, grade 1–2, with negative or focal LVSI. Intermediate risk included stage IB endometrioid carcinoma, grade 1–2, with negative or focal LVSI, and stage IA endometrioid carcinoma, grade 3, with negative or focal LVSI. High-intermediate risk included stage I endometrioid carcinoma with substantial LVSI regardless of grade or depth of invasion, stage IB grade 3 endometrioid carcinoma regardless of LVSI status, and stage II endometrioid carcinoma. Available H&E slides from LVSI-positive cases were re-reviewed specifically for this secondary risk-group analysis to distinguish focal from substantial LVSI; the original binary LVSI variable was retained for all primary and previously specified analyses. CD163-positive TAM density was compared using the Kruskal–Wallis test, and high-TAM proportions were compared using Pearson’s χ² test. High TAM infiltration was defined as >55 cells/HPF. Because the cohort was restricted to stage I–II endometrioid carcinoma, no patient met the high-risk or advanced/metastatic definitions in this clinicopathologic classification. LVSI, lymphovascular space invasion; TAM, tumor-associated macrophage; HPF, high-power field; IQR, interquartile range.

Supplementary Figure 1 Exploratory disease-free survival according to combined MMR–TAM phenotypes **(A)** Kaplan–Meier estimates comparing patients with the dMMR/high-TAM phenotype with all remaining MMR–TAM phenotypes. The dMMR/high-TAM group included 44 patients with 11 DFS events, whereas the remaining phenotypes included 192 patients with 15 events. Estimated 3-year DFS was 78.4% and 92.3%, respectively (log-rank P = 0.018). **(B)** Kaplan–Meier estimates across the four predefined phenotypes: pMMR/low TAM, pMMR/high TAM, dMMR/low TAM, and dMMR/high TAM. DFS events occurred in 6/101, 6/71, 3/20, and 11/44 patients, respectively; the global log-rank P value was 0.021. Numbers at risk are shown below the curves. These analyses were exploratory and do not establish statistical interaction or incremental prognostic value of TAM infiltration beyond MMR status. DFS, disease-free survival; MMR, mismatch repair; pMMR, proficient mismatch repair; dMMR, deficient mismatch repair; TAM, tumor-associated macrophage.
