# Supplementary material for: CD163-positive macrophage enrichment is associated with mismatch repair deficiency and adverse pathologic features in early-stage endometrioid endometrial carcinoma: a retrospective cohort study
Source: Front Oncol. 2026 Oct 2;16:1954599. doi: 10.3389/fonc.2026.1954599 (PMC13671934; doi:10.3389/fonc.2026.1954599)
Supplement: Supplementary file 1 [file Table1.docx]

**Supplementary Table S1. Baseline characteristics according to CD163+ TAM infiltration status**

| **Variable** | **Low TAM (n = 121)** | **High TAM (n = 115)** | **Test statistic** | **P value** |
| --- | --- | --- | --- | --- |
| Age, years | 56 (49–64) | 58 (51–66) | Z = 1.53 | 0.126 |
| Postmenopausal status | 75 (62.0) | 76 (66.1) | χ² = 0.43 | 0.512 |
| Body mass index, kg/m² | 26.9 ± 4.7 | 27.4 ± 4.8 | t = 0.81 | 0.420 |
| Hypertension | 39 (32.2) | 44 (38.3) | χ² = 0.94 | 0.332 |
| Diabetes mellitus | 20 (16.5) | 23 (20.0) | χ² = 0.48 | 0.490 |
| Tumor size ≥3 cm, n/N (%) | 48/117 (41.0) | 59/111 (53.2) | χ² = 3.36 | 0.067 |
| FIGO stage IA | 74 (61.2) | 56 (48.7) |  |  |
| FIGO stage IB | 29 (24.0) | 33 (28.7) | χ² = 4.05 | 0.132 |
| FIGO stage II | 18 (14.9) | 26 (22.6) |  |  |
| Grade 1 | 52 (43.0) | 32 (27.8) |  |  |
| Grade 2 | 49 (40.5) | 48 (41.7) | χ² = 8.72 | 0.013 |
| Grade 3 | 20 (16.5) | 35 (30.4) |  |  |
| Deep myometrial invasion ≥50% | 36 (29.8) | 58 (50.4) | χ² = 10.52 | 0.001 |
| LVSI positive | 13 (10.7) | 28 (24.3) | χ² = 7.60 | 0.006 |
| dMMR status | 20 (16.5) | 44 (38.3) | χ² = 14.09 | <0.001 |
| Adjuvant radiotherapy, n/N (%) | 28/120 (23.3) | 38/113 (33.6) | χ² = 3.04 | 0.081 |
| Adjuvant chemotherapy, n/N (%) | 14/120 (11.7) | 25/113 (22.1) | χ² = 4.57 | 0.033 |
| Follow-up, months | 48 (36–60) | 49 (37–59) | Z = 0.41 | 0.682 |

Note: Values are n (%), n/N (%), median (IQR), or mean ± SD. Percentages for variables with missing data were calculated using available-case denominators. LVSI, lymphovascular space invasion; TAM, tumor-associated macrophage.
