# Supplementary material for: CD163-positive macrophage enrichment is associated with mismatch repair deficiency and adverse pathologic features in early-stage endometrioid endometrial carcinoma: a retrospective cohort study
Source: Front Oncol. 2026 Oct 2;16:1954599. doi: 10.3389/fonc.2026.1954599 (PMC13671934; doi:10.3389/fonc.2026.1954599)
Supplement: Supplementary file 2 [file Table2.docx]

**Supplementary Table S2. Clinicopathologic features across combined MMR–TAM phenotypes**

| **Variable** | **pMMR/low TAM (n = 101)** | **pMMR/high TAM (n = 71)** | **dMMR/low TAM (n = 20)** | **dMMR/high TAM (n = 44)** | **P value** |
| --- | --- | --- | --- | --- | --- |
| Age, years | 55 (49–64) | 57 (50–65) | 58 (51–66) | 60 (53–68) | 0.096 |
| FIGO stage II | 13 (12.9) | 18 (25.4) | 5 (25.0) | 8 (18.2) | 0.182 |
| Grade 3 histology | 16 (15.8) | 18 (25.4) | 4 (20.0) | 17 (38.6) | 0.026 |
| Tumor size ≥3 cm, n/N (%) | 39/98 (39.8) | 36/69 (52.2) | 9/19 (47.4) | 23/42 (54.8) | 0.284 |
| Deep myometrial invasion ≥50% | 28 (27.7) | 32 (45.1) | 8 (40.0) | 26 (59.1) | 0.003 |
| LVSI positive | 9 (8.9) | 16 (22.5) | 4 (20.0) | 12 (27.3) | 0.024 |
| Composite adverse pathologic feature | 26 (25.7) | 35 (49.3) | 7 (35.0) | 21 (47.7) | 0.007 |
| Adjuvant radiotherapy, n/N (%) | 22/100 (22.0) | 24/70 (34.3) | 6/20 (30.0) | 14/43 (32.6) | 0.308 |
| Adjuvant chemotherapy, n/N (%) | 11/100 (11.0) | 14/70 (20.0) | 3/20 (15.0) | 11/43 (25.6) | 0.148 |
| DFS event during follow-up | 6 (5.9) | 6 (8.5) | 3 (15.0) | 11 (25.0) | 0.007 |

Note: Values are n (%), n/N (%), or median (IQR). Percentages for variables with missing data were calculated using available-case denominators. P values compare the four combined MMR–TAM phenotypes and were obtained using χ² tests, Fisher’s exact tests, or Monte Carlo exact tests when sparse cells were present. DFS, disease-free survival; LVSI, lymphovascular space invasion; TAM, tumor-associated macrophage.
