# Supplementary material for: CD163-positive macrophage enrichment is associated with mismatch repair deficiency and adverse pathologic features in early-stage endometrioid endometrial carcinoma: a retrospective cohort study
Source: Front Oncol. 2026 Oct 2;16:1954599. doi: 10.3389/fonc.2026.1954599 (PMC13671934; doi:10.3389/fonc.2026.1954599)
Supplement: Supplementary file 3 [file Table3.docx]

**Supplementary Table S3. Sensitivity analyses**

| **Sensitivity analysis** | **Effect estimate (95% CI)** | **P value** |
| --- | --- | --- |
| Association between dMMR and high TAM after excluding stage II disease | OR 2.12 (1.08–4.15) | 0.029 |
| Association between dMMR and high TAM after additional adjustment for adjuvant therapy | OR 2.18 (1.13–4.23) | 0.020 |
| Association between CD163+ TAM density per 10 cells/HPF and composite adverse pathologic feature | OR 1.11 (1.02–1.22) | 0.018 |
| Association between high TAM and composite adverse pathologic feature in stage I-only patients | OR 1.69 (0.99–2.89) | 0.054 |
| Association between dMMR/high TAM phenotype and DFS after adjustment for adjuvant therapy | HR 2.04 (0.93–4.52) | 0.075 |
| Association between dMMR/high TAM phenotype and DFS after excluding patients with follow-up <12 months | HR 2.31 (1.02–5.22) | 0.045 |

Note: Sensitivity analyses were performed to examine the robustness of the primary association and exploratory DFS findings. DFS, disease-free survival; HR, hazard ratio; OR, odds ratio; TAM, tumor-associated macrophage.
