# Supplementary material for: CD163-positive macrophage enrichment is associated with mismatch repair deficiency and adverse pathologic features in early-stage endometrioid endometrial carcinoma: a retrospective cohort study
Source: Front Oncol. 2026 Oct 2;16:1954599. doi: 10.3389/fonc.2026.1954599 (PMC13671934; doi:10.3389/fonc.2026.1954599)
Supplement: Supplementary file 4 [file Table4.docx]

**Supplementary Table S4. Data completeness and model diagnostics**

| **Item** | **Available data** | **Missing data** | **Assessment** |
| --- | --- | --- | --- |
| MMR status | 236/236 | 0 (0.0) | Complete |
| CD163+ TAM count | 236/236 | 0 (0.0) | Complete |
| CD163+ TAM category | 236/236 | 0 (0.0) | Complete |
| Tumor size | 228/236 | 8 (3.4) | Low missingness; available-case analysis |
| LVSI status | 236/236 | 0 (0.0) | Complete |
| Adjuvant treatment data | 233/236 | 3 (1.3) | Low missingness; available-case analysis |
| Follow-up status | 236/236 | 0 (0.0) | Complete |
| Logistic regression collinearity | Not applicable | Not applicable | Maximum VIF = 1.74 |
| Cox proportional hazards assumption | Not applicable | Not applicable | Global Schoenfeld P = 0.72 |
| CD163+ TAM interobserver agreement | Not applicable | Not applicable | ICC = 0.88 |
| MMR status interobserver agreement | Not applicable | Not applicable | κ = 0.94 |

Note: Missing values were not imputed. Descriptive summaries used available-case denominators for variables with missing data, and regression models were performed using complete-case analysis. VIF, variance inflation factor; ICC, intraclass correlation coefficient; LVSI, lymphovascular space invasion; TAM, tumor-associated macrophage.
