# Supplementary material for: CD163-positive macrophage enrichment is associated with mismatch repair deficiency and adverse pathologic features in early-stage endometrioid endometrial carcinoma: a retrospective cohort study
Source: Front Oncol. 2026 Oct 2;16:1954599. doi: 10.3389/fonc.2026.1954599 (PMC13671934; doi:10.3389/fonc.2026.1954599)
Supplement: Supplementary file 5 [file Table5.docx]

**Supplementary Table S5. CD163-positive TAM infiltration across ESGO–ESTRO–ESP clinicopathologic risk groups**

| ESGO–ESTRO–ESP risk group | Patients, n (%) | CD163+ TAM density, cells/HPF, median (IQR) | High CD163+ TAM infiltration, n (%) |
| --- | --- | --- | --- |
| Low risk | 98 (41.5) | 46 (28–72) | 38 (38.8) |
| Intermediate risk | 66 (28.0) | 55 (33–80) | 32 (48.5) |
| High-intermediate risk | 72 (30.5) | 63 (40–91) | 45 (62.5) |
| Overall | 236 (100.0) | 55 (31–84) | 115 (48.7) |
| Global P value | — | 0.016 | 0.009 |

**Note:** Risk groups were assigned according to the 2021 ESGO–ESTRO–ESP clinicopathologic framework for patients with unknown molecular classification. Low risk included FIGO 2009 stage IA endometrioid carcinoma, grade 1–2, with negative or focal LVSI. Intermediate risk included stage IB endometrioid carcinoma, grade 1–2, with negative or focal LVSI, and stage IA endometrioid carcinoma, grade 3, with negative or focal LVSI. High-intermediate risk included stage I endometrioid carcinoma with substantial LVSI regardless of grade or depth of invasion, stage IB grade 3 endometrioid carcinoma regardless of LVSI status, and stage II endometrioid carcinoma. Available H&E slides from LVSI-positive cases were re-reviewed specifically for this secondary risk-group analysis to distinguish focal from substantial LVSI; the original binary LVSI variable was retained for all primary and previously specified analyses. CD163-positive TAM density was compared using the Kruskal–Wallis test, and high-TAM proportions were compared using Pearson’s χ² test. High TAM infiltration was defined as >55 cells/HPF. Because the cohort was restricted to stage I–II endometrioid carcinoma, no patient met the high-risk or advanced/metastatic definitions in this clinicopathologic classification. LVSI, lymphovascular space invasion; TAM, tumor-associated macrophage; HPF, high-power field; IQR, interquartile range.
